# Family functioning and personal growth in Italian caregivers living with a family member affected by schizophrenia: Results of an add-on study of the Italian network for research on psychoses

**DOI:** 10.3389/fpsyt.2022.1042657

**Published:** 2023-01-13

**Authors:** Rita Roncone, Laura Giusti, Valeria Bianchini, Massimo Casacchia, Bernardo Carpiniello, Eugenio Aguglia, Mario Altamura, Stefano Barlati, Antonello Bellomo, Paola Bucci, Giammarco Cascino, Carmen Concerto, Andrea Fagiolini, Carlo Marchesi, Alessio Maria Monteleone, Federica Pinna, Alberto Siracusano, Silvana Galderisi

**Affiliations:** ^1^Psychiatry Unit, Department of Life, Health and Environmental Sciences, University of L’Aquila, L’Aquila, Italy; ^2^Department of Medical Sciences and Public Health, University of Cagliari, Cagliari, Italy; ^3^Psychiatry Unit, Department of Clinical and Experimental Medicine, University of Catania, Catania, Italy; ^4^Department of Clinical and Experimental Medicine, University of Foggia, Foggia, Italy; ^5^Psychiatric Unit, Department of Clinical and Experimental Sciences, Brescia University, Brescia, Italy; ^6^Department of Psychiatry, University of Campania “Luigi Vanvitelli”, Naples, Italy; ^7^Department of Medicine, Surgery and Dentistry “Scuola Medica Salernitana,” Section of Neuroscience, University of Salerno, Salerno, Italy; ^8^Department of Molecular Medicine, University of Siena, Siena, Italy; ^9^Psychiatric Unit, Department of Medicine and Surgery, University of Parma, Parma, Italy; ^10^Psychiatry and Clinical Psychology Unit, Department of Systems Medicine, Tor Vergata University of Rome, Rome, Italy

**Keywords:** burden of illness, schizophrenia, family caregivers, family functioning, personal growth

## Abstract

To date, the role of family members in caring for relatives affected by schizophrenia has focused largely on the negative aspects of impact of the illness. The present study aimed to: (1) assess family functioning and burden of care in caregivers living in Northern, Central, and Southern Italy who looked after subjects affected by chronic schizophrenia; (2) evaluate the relationship between aspects of family functioning and burden of care, in particular personal growth (PG) of caregivers; and (3) identify variables capable of affecting PG of caregivers. A total of 136 caregivers (mean length of illness of family member more than 20 years) were recruited from 9 Italian research sites and evaluated in terms of “positive” family functioning–problem-solving, communication skills and personal goals Family Functioning Questionnaire (FFQ), burden of care, and PG Family Problems Questionnaire (FPQ). Caregivers reported an overall good family functioning with a relatively low objective and subjective burden of care. The latter was positively correlated with length of illness, with women showing a higher subjective burden than men. Reduced problem-solving skills and ability of each family member to pursue personal goals were both associated with reduced objective and subjective burden which, conversely, were both increased by inadequate support and scarce positive comments from relatives and friends. Approximately 50% of caregivers stated that “they had learned something positive from the situation,” highlighting a statistically higher proportion of caregivers in southern Italy than in northern and central Italy. Caregivers’ PG was associated with good family functioning, adequate professional support, and positive comments. PG also seemed to be positively influenced by support from relatives and friends (O.R. 14.306). The numerous challenges and positive aspects associated with caregiving should be duly acknowledged by mental health services and integrated into routine clinical assessment and intervention framework.

## 1. Introduction

Relatives in Italy are closely involved in caring for family members with physical and mental disorders (1). Indeed, whilst families have tended to become increasingly smaller, they continue to maintain close ties, with adolescents not leaving the parental home until relatively late in life (2). A prolonged presence of grown children in their parents’ home is considered indicative of a united family, whereas Americans and Western Europeans tend to opt for greater individualism and inter-generational independence (3).

Suffering and distress are healthy, normal reactions when someone close is affected by schizophrenia. In early psychosis, parents experience a grieving process in which they try to reconcile past hopes and dreams for their child with more realistic ones (4). In line with the stress theory, increased duration of illness and care negatively impact on caregivers who experience high levels of stress, perceiving an enforced restriction on their ability to function effectively or having most of their day taken up by caring for their ill relative (5–10). In addition, functioning difficulties in the family may lead to increased stress in caregivers (11–16), to lack of appropriate strategies to treat symptoms manifested by the sick relative, and financial problems (17). Conversely, the mental health of caregivers may be preserved by working outside the home, generating income, maintaining activities unrelated to their role as caregiver and pursuing personal life goals (5).

Emphasis on the role of the family in caring for people affected by schizophrenia has generally focused on the reciprocal negative impact produced by family atmosphere on the ill person (18, 19) and by burden of care on relatives (5, 17, 20–24).

Models of negative and positive caregiving experiences were studied by Campos et al. (25) taking into account the caregiver’s perceptions of difficulties, satisfaction, and coping. A positive caregiving experience was frequently justified based on perception of sources of intrapersonal satisfaction, i.e., caregivers fulfilled their role and duties and attributed a specific meaning to the caregiving experience (sense of life, maturation, growth, development of new skills, self-esteem).

Evidence of the effectiveness of psychoeducational interventions involving family members aimed at mitigating effects of the disease (26–29) allowed the concept of “schizophrenogenic mother” and negative family influence to be quelled. Accordingly, the next step to be taken in studying “the family and mental illness” should focus on evaluating the efforts of each family member “in honestly doing their best” to cope with the grief and practical difficulties related with their relative’s illness.

Despite the finding of a possible intermediate severity of impairment in cognitive functions (30) amongst first-degree relatives of schizophrenia patients, these seem to display a good degree of accuracy in assessing real-life functioning of their mentally ill family members (31). Identification of the strengths and positive aspects of caregiver experiences represents an interesting, and relatively unexploited, area of study (32, 33). Positive experiences referred by caregivers highlighted benefits such as increased sensitivity to people with disabilities, clarity over priorities in life, and a greater sense of inner strength (32, 34).

Given the relevance of roles assumed by families in the context of community psychiatric care in looking after relatives affected by mental disorders (1), together with a growing interest in studying positive caregiving experiences in the area of schizophrenia, the present study was designed to investigate factors capable of positively influencing the experience of Italian caregivers devoted to the care of family members diagnosed with schizophrenia.

The study aimed to: (1) assess family functioning and burden of care in caregivers living with subjects affected by chronic schizophrenia with specific focus on their family role and distribution throughout the three main geographical areas of the country (Northern, Central, and Southern Italy); (2) evaluate the relationship between aspects of family functioning and burden of care; and (3) identify variables capable of promoting a deeper acquisition of the meaning of life following the experience of living with someone affected by a severe mental disorder.

We hypothesized that good family functioning was indirectly associated with burden of care, and that the personal growth (PG) of caregivers, perceiving positive experience in assisting a person affected by severe mental illness could be a relevant variable in the caregiving process.

## 2. Materials and methods

### 2.1. Subjects

The study, designed as an add-on study investigating family characteristics, was conducted on a sample of 136 caregivers of subjects affected by schizophrenia initially enrolled in a study undertaken by the Italian Network for Research on Psychoses (NIRP) (35). The inclusion and exclusion criteria of the affected subjects have been described in a previous paper (35). They were clinically comparable, in terms of the severity of illness and psychopathological features and showed a modest degree of functional impairment (35). Recruitment took place from March 2012 to September 2013. The sample included unaffected family members recruited as the main caregiver, the so-called “face-to-face” relatives. The latter were preferably one of the parents, wife or husband, or sibling of the ill family member, i.e., the person most frequently and closely in contact with the affected person and/or was considered the “main caregiver.”

Nine research sites (out of 28 involved in the NIRP, 32%) took part in the study, the university psychiatric clinics of Brescia, Cagliari, Catania, Foggia, L’Aquila, Parma, Roma “Tor Vergata,” Salerno, and Siena. In consideration of the regional cultural diversity that characterizes Italy, caregivers were grouped according to three macro-areas: Northern Italy (two centers), Central Italy (two centers), and Southern Italy (five centers, including the islands of Sicily and Sardinia). A 1-day training program was organized to illustrate the rationale of this add-on research and two psychiatrists/psychologists joined from each participating center.

Socio-demographic and clinical data of users were extracted from the main database of the study (35). All subjects signed a written informed consent after receiving a comprehensive explanation of the study procedures and goals. The study has been conducted in accordance with the principles of the Declaration of Helsinki (59th World Medical Association General Assembly; October 2008). Approval of the study protocol was obtained from the Ethics Committees of the participating centers.

### 2.2. Assessment tools

#### 2.2.1. Caregiver evaluation tools

All participants completed a form to provide socio-demographic data and the following evaluation tools were administered.

#### 2.2.2. Family functioning

Family functioning was assessed using the Family Functioning Questionnaire (FFQ) (36). Developed to assess the pattern of family functioning, at the center of psychoeducational family interventions, the questionnaire consists of 24 items measuring the following 3 dimensions:

(1) Problem solving (eight items), referred to the six steps of structured problem-solving: identify the problem or the objective, list possible alternative solutions, discuss the positive and negative aspects of each proposal, choose the best (or better, a satisfying, and realistic solution), plan the solution, check and review implementation and planning;
(2) Communication skills (eight items), concerning the expression of positive and negative feelings, making of requests and active listening skills (probing questions, a summary of what has been understood), and
(3) Personal Goals (eight items), defined as the ability of each family member to identify everyday personal goals (not linked to subject care). Responses range from 1 “never” to 4 “always.” Higher scores are indicative of healthier functioning.

The items are evaluated on a four-point Likert scale; a high score is associated with better family functioning (range 24–96). The scale was originally developed and standardized in the Italian population and has demonstrated good internal consistency (Cronbach’s alpha coefficient ranges from 0.75 to 0.84 for the three dimensions) and test–retest reliability (Pearson’s r correlation coefficient ranges from 0.75 to 0.60) (36). Internal consistency for the FFQ in our sample was high (Cronbach’s a = 0.88).

#### 2.2.3. Burden of care

The version of the Family Problem Questionnaire (FPQ) (37) used in this study consists of 39 items (38). Items are evaluated on a four-point Likert scale. The self-administered instrument investigates five conceptual dimensions: (1) objective burden (thirteen items, range 13–52), as the impact on daily activities/social life; (2) subjective burden (six items, range 6–24), as the impact on caregiver wellbeing, distress over the condition of the affected family member, concern for the future; (3) professional support received (four items, range 4–16); (4) support from relatives and friends (three items, range 3–12); (5) positive attitudes (four items, range 4–16), and (6) negative attitudes (criticism, hostility) toward the affected relative (two items, range 2–8).

If the household comprises children below the age of 12 years, the respondent is asked to assess impact of the situation on the children (two items, range 2–8) as well as repercussions on their social life or psychological wellbeing.

Economic burden (four items) is defined as direct costs (professional, alternative medicine, drugs, and all non-reimbursable expenses) incurred by the family and loss of income by family members forced to reduce their working hours or to take a lower paid job.

A single item measured caregiver’s PG [item 31: “*All things considered, I learned something positive from this situation (for example, to understand myself and others better”*].

Higher scores are associated with a higher burden of care, scarce support from professionals, relatives and friends, and negative communication.

### 2.3. Data analysis

Parametric and non-parametric statistics were utilized in data analysis. Chi-squared test and *t*-test for independent samples were conducted to examine the differences in variables relating to sociodemographics and family functioning and burden of care, as measured by FFQ and FPQ. A one-way ANOVA test was conducted to analyze effect of the family role (parent/sibling) and geographical area (Northern, Central, and Southern Italy) on family functioning and burden of care.

A correlation analysis (r-Pearson) was conducted to verify relationships among caregivers’ PG score, the three-dimensions (problem-solving, communication, and personal goals) of family functioning (FFQ), the six-subscales (objective burden, subjective burden, professional support, support from relatives and friends, positive comments, negative comments) of burden of care, as measured by FPQ, and patient’s length of illness and caregiver’s years of education.

Multinominal logistic regression analyses were conducted to identify variables capable of influencing caregiver’s PG. The dependent variable Caregiver’s PG (“*All things considered, I learned something positive from this situation, for example, to understand myself and others better*”) was coded 1 = very much; 2 = quite a lot; 3 = only a little; 4 = not at all. Independent variables in the model included age of caregivers and patients, patients’ length of illness, the three dimensions of the FFQ (problems solving, communication, and personal goals) scores, and four subscales of the FPQ (objective and subjective burden, professional support, and family and friends support). With regard to our model, the selection of independent variables was based prevalently on previous research findings related to the caregiving experience. “Age of caregivers” was included as a potential predictor based on the finding that lower age of caregivers seemed to predict better stress management (15, 39). We also selected “subject age” since poor psychological wellbeing amongst family members was associated with the presence of younger affected relatives with earlier age of onset of the disease (40). “Subjects’ length of illness” was identified as a variable influencing burden of care, i.e., the longer the duration of illness, the heavier the burden of care (41–43). In our model, selection of the three dimensions of family functioning were based on the assumption that good family functioning could potentially predict caregiver’s PG, together with reduced burden of care and excellent support from professionals, relatives and friends (44).

We conducted odds ratios with 95% confidence intervals for nominal regression analysis. Statistical analyses were performed using SPSS 26.0 (SPSS Inc., Chicago, IL, USA).

## 3. Results

Out of a total of 164 potentially eligible caregivers, assessments received from 136 (83%) were deemed valid. Twenty-eight assessments were excluded from analysis as they were incomplete or lacking accurate completion of one of the two caregiver evaluation tools. Family members completed both self-administered family instruments in a mean of 20-min.

The main socio-demographic and clinical data of affected subjects and their caregivers are illustrated in Table 1.

**TABLE 1:** Demographic characteristics of user and caregiver respondents (*n* = 136).

Users	Women (no = 36; 26.5%)	Men (no = 100, 73.5%)	
Age, mean (SD)	46.7 (11.0)	43.1 (8.8)	
Education, year, mean (SD)	11.9 (3.8)	11.7 (3.0)	
Length of illness, years (SD)	22.5 (9.9)	21.5 (9.1)	
**Caregivers**	**Women (no = 85, 62.5%)**	**Men (no = 51, 37.5%)**	
**Role in the family (%)**			
Parents	60 (70.6)	36 (70.6)	
Siblings	15 (17.6)	10 (19.6)	
Children	4 (4.7)	–	
Partner	6 (7.1)	2 (3.9)	
Other	–	3 (5.9)	
**Age, mean (SD)**			
Parents	69.7 (9.0)	70.6 (6.9)	
Siblings	55.9 (10)	50.2 (13.6)	
Children	44.0 (8.2)	–	
Partner	42.5 (12.7)	45.2 (13.3)	
Other	–	56.2 (5.4)	
**Education, years mean (SD)**	9.9 (3.5)	10.5 (3.9)	
**Work status (%)**			
Competitive work	20 (23.5)	14 (27.5)	
Supported work	2 (2.4)	2 (3.9)	
Student	–	1 (2.0)	
Housewife	17 (20.0)	–	
Unemployed	8 (9.4)	5 (9.8)	
Retired	30 (35.3)	27 (52.9)	
Disability pensioner	8 (9.4)	2 (3.9)	
**Geographical area of residence (%)**			
Northern Italy	5 (35.7)	9 (64.3)	
Central Italy	12 (85.7)	2 (14.3)	
Southern Italy	68 (63.0)	40 (37.0)	
			Chi-square test: 7.515 *p* = 0.023

The sample included subjects affected by schizophrenia, mean aged 44.07 (SD 9.5, range 24–69), with average illness duration of 21.8 years (SD 9.2, range 5–49). Males represented three-quarters of the sample. No statistically significant differences based on gender were found for mean age, years of education, and length of illness.

Women represented more than 60% of the caregiver sample. No statistically significant differences based on gender were detected for mean age (women 66.0 SD 12.1, men 65.3 SD 12.1) and years of education. The sample was characterized by being an elderly relative (mainly mothers and fathers) and low level of education, with approximately 75% not being in current competitive employment. The mean number of family members was 3 (SD 1; range 2–9).

One hundred and eight (79.4%) caregivers were from Southern Italy, 14 (10.3%) from Northern Italy, and 14 (10.3%) from Central Italy. Based on geographical location of residence, a statistically significant different distribution of caregivers was found among participants. Female caregivers were distributed increasingly throughout Central and Southern Italy, whereas males comprised approximately 60% of interviewed caregivers in the Northern area (Table 1).

### 3.1. Family functioning

Total FFQ score corresponded to 66.19 (SD 10.9; range 37–93), showing an overall good family functioning amongst caregivers included in our sample. FFQ mean scores for gender, roles, and location are shown in Table 2. No statistically significant differences based on gender were found in either total score or the three dimensions assessed. Statistically significant higher scores were found for “communication” with regard to marital vs. filial roles (ANOVA: *F* = 3.039; *p* = 0.031; *post hoc* Bonferroni’s: 0.93750; *p* = 0.019) and for caregivers living in Northern compared to central Italy (ANOVA: *F* = 3.179; *p* = 0.045; *post hoc* Bonferroni’s: 0.47321; *p* = 0.049), showing better communication skills. Statistically significant higher scores were found for “personal goals” for caregivers living in Southern compared to Central Italy (ANOVA: *F* = 4.111; *p* = 0.019; *post hoc* Bonferroni’s: 0.36723; *p* = 0.019), displaying better skills in pursuing individual goals.

**TABLE 2:** Total mean scores at dimensions of the family functioning questionnaire (FFQ) according to gender, family roles, and location of caregivers (higher scores indicate better functioning).

	Problem-solving	Communication	Personal goals
**Gender**			
Women	2.66 (0.63)	2.90 (0.53)	2.64 (0.50)
Men	2.75 (0.61)	2.91 (0.50)	2.75 (0.42)
**Roles**			
Parents	2.70 (0.57)	2.90 (0.47)	2.65 (0.48)
Siblings	2.67 (0.66)	2.89 (0.58)	2.78 (0.49)
Children	2.09 (1.04)	2.31 (0.96)*	2.56 (0.31)
Partner	2.82 (0.68)	3.25 (0.38)*	2.79 (0.49)
**Geographical area**			
Northern Italy	2.81 (0.73)	3.08 (0.53)^§^	2.76 (0.50)
Central Italy	2.31 (0.63)	2.61 (0.65)^§^	2.34 (0.56)^#^
Southern Italy	2.73 (0.59)	2.92 (0.52)	2.71 (0.44)^#^

*Statistically significant higher scores for marital compared to filial role; *p* < 0.05. ^§^Statistically significant higher scores for caregivers living in Northern Italy than those living in central Italy; *p* < 0.05. ^#^Statistically significant higher scores in “personal goals” for caregivers living in Southern Italy than those living in Central Italy; *p* < 0.05.

### 3.2. Burden of care

#### 3.2.1. Objective burden

Total mean FPQ score for objective burden dimension was 1.71 (SD 0.59; range 1–3.42), showing a low objective burden in the sample of caregivers (Table 3). No statistically significant differences were found based on gender, role, and location. To better describe the socio-economic family context, Table 4 illustrates the six items respondents were asked to refer to over the last 2 months (items 10, 14, 16) or the previous year (items 15, 17, 18). Answers provided revealed how approximately 35% had never made weekend trips or taken holidays. In one-third of the sample, no members of the household in employment, whilst in one-fifth, the caregiver and affected person lived alone. Approximately one-fifth of parents reported feelings of guilt caused by the illness of their son/daughter.

**TABLE 3:** Total mean scores at dimensions of the family problem questionnaire (FPQ) according to gender, family roles, and location (higher scores indicate higher burden and worse evaluation).

	Objective burden	Subjective burden	Support received from professionals	Support received from relatives and friends	Positive attitudes	Negative attitudes
**Gender**						
Women	1.77 (0.60)	2.00 (0.54)	1.83 (0.56)	2.62 (0.61)	1.63 (1.49)	1.68 (1.10)
Men	1.61 (0.57)	1.73 (0.53)	1.75 (0.47)	2.45 (0.67)	1.46 (1.50)	2.01 (1.48)
		*t*-test: −2.825; *p* = 0.005				
**Roles**						
Parents	1.72 (0.62)	1.93 (0.58)	1.80 (0.51)	2.59 (0.66)	1.56 (1.49)	1.69 (1.17)
Siblings	1.66 (0.56)	1.82 (0.47)	1.92 (0.60)	2.58 (0.52)	1.52 (1.27)	2.26 (1.49)
Children	1.83 (0.42)	1.83 (0.30)	1.81 (0.80)	2.66 (0.47)	1.00 (2.00)	1.75 (0.50)
Partner	1.64 (0.60)	1.85 (0.71)	1.53 (0.41)	2.33 (0.61)	2.00 (1.92)	1.93 (1.78)
**Geographical area^§^**						
Northern Italy	1.59 (0.66)	1.84 (0.45)	1.98 (0.76)	2.90 (0.82)	2.21 (1.42)	2.03 (1.63)
Central Italy	1.75 (0.42)	1.96 (0.49)	1.58 (0.49)	2.33 (0.55)*	1.78 (1.67)	1.39 (0.44)
Southern Italy	1.72 (0.60)	1.90 (0.57)	1.81 (0.50)	2.52 (0.61)*	1.46 (1.46)	1.83 (1.28)

^§^Caregivers from Central and Southern Italy reported higher levels of support from relatives and friends than caregivers in Northern Italy. *p = 0.05.

**TABLE 4:** Selected items relating to objective burden in FPQ.

Family problems questionnaire, FPQ (SD)		
**Objective burden (%)**		
Item 10. *Taking a weekend trip or a weekend away*	Never done	47 (34.6)
Item 14. *Neglecting other family members*	Nobody lives with us	26 (19.1)
Item 15. ***Over the last year*,** *taking a holiday*	Never done	51 (37.5)
Item 16. (only relative) *Feeling guilty for the illness of S. Missing 32 (23.5%)*	Very much, quite a lot	24 (17.6)
Item 17. ***Over the last year***, *impact on work of one of the family members*		
Item 18. **Over *the last year***, *need to work more by one of the family members*	Nobody works	46 (33.8)

#### 3.2.2. Subjective burden

In this dimension, a total mean score of 1.90 (SD 0.55; range-1–3.50) emphasized a relatively low subjective burden in the caregiver sample, with a statistically significant higher burden for women than men (Table 3). No statistically significant differences were found based on role and location. The most distressing complaint was related to item 24 (“*When I think about how S. was before he got sick and how he is now, I feel great pain*”) (score 4, “very, very much” *n* = 22, 16.2%).

#### 3.2.3. Professional support

A total mean score of 1.80 (SD 0.50; range 1–3.50) highlighted a relatively satisfying amount of professional support. No statistically significant differences were found based on gender, family role, and location (Table 3). Approximately 90% of caregivers (*n* = 112, 89.1%) reported strong support from professionals (score 1–2 on item 4), associated with the conviction that doctors and other professionals would provide immediate assistance in the case of an emergency involving the affected person.

#### 3.2.4. Support from relatives and friends

A total mean score of 2.56 (SD 0.64; range 1–4) revealed modest satisfaction in the support provided by relatives and friends (Table 3). The highest concern was related to anxiety that, with the exception of family members, there was no-one else to take care of the ill relative (score 3–4 on item 7, *n* = 101, 73.7%). No statistically significant differences were found based on gender or family role. Caregivers living in Northern Italy showed a lower satisfaction with support received from relatives and friends compared to those living in Central (*p* = 0.018; 95% CI 0.1013–1.0416) and Southern Italy (*p* = 0.034; 95% CI −1.0416–0.1013) (Table 3).

#### 3.2.5. Positive attitudes toward the affected subject

Items 25–28 identified positive comments (scoring 1 or 2 on the four items) relating to the person being cared for with regard to his/her “*sensitivity to care about the problems of others*” (45.9%), “*cooperation with people trying to help him/her*” (44.4%), “*special talents or abilities*” (37.8%), “*giving practical help in the household*” (31.1%).

Thirty-eight (74,5%) of the 51 caregivers who reported how the relative they cared for had “*special talents or abilities*” provided detailed answers and expressed emotional warmth. They referred to unique talents or abilities including “intelligence,” “generosity,” “sympathy,” “goodness,” “altruism,” and “creativity.” Practical skills (making pizza, playing guitar, painting, taking pictures, being a good electrician, doing handicraft works) were listed among the examples. Explicit appreciation was expressed irrespective of gender of the affected subject.

With regard to the “positive attitudes” dimension, a cut-off of 8 was considered expression of “positive comments and warmth,” with 31.6% of our sample (*n* = 43) deemed highly competent in this area. No statistically significant differences were detected based on gender, roles, and geographical area (Table 3).

#### 3.2.6. Negatives attitudes toward the affected subject

In this dimension, the two items included in the questionnaire aimed to identify “criticism” and “hostility” reported by the Expressed Emotion index, respectively. In our sample, the first item (item 29), related to the exhibition of deliberately strange behaviors, a paradigmatic expression of “criticism,” 81 caregivers (59.6%) denied any intention to annoy their affected person “on purpose.” The remaining caregivers reported how this occurred “sometimes” (32 subjects, 23.5%) or “often” (8 subjects, 5.9%), whilst 15 (11%) stated how the affected person never displayed any strange behaviors. No statistically significant differences were found based on gender, role, location of the affected person.

At item 30, the second item of this dimension examining readiness of the caregiver to disconnect from the affected person, typically viewed as an expression of “hostility,” 95 caregivers (70%) rejected the idea, 31 (22.5%) answered they had thought about doing so “sometimes,” 8 (6%) “often,” whilst only 2 caregivers (1.5%) confirmed they thought about doing so “every day.”

Caregivers’ gender was not associated with a statistically significant difference at either item in reporting negative attitudes in family members. Likewise, male gender did not produce a higher degree of negativity in caregivers with regard to expectation of a more active social role of their affected member. Partners displayed higher degrees of hostility. With regard to family roles, a statistically significant difference was found in distribution of increased hostility (scoring 3–4 on item 30) amongst partners (50%) compared to siblings (20%), parents (3.1%), and children (0%) (chi-square: 21.194; *p* = 0.012).

Eighteen caregivers (13.2%) scoring 3 or 4 at item 29 and/or item 30 were deemed “highly critical-hostile.”

#### 3.2.7. Impact of the situation on minors

Only 11 caregivers (8.1% of the sample) answered the two items relating to the dimension assessing impact of the affected subject’s presence on their children’s social life or psychological wellbeing. They indicated a moderately negative influence, with two caregivers (1.4%) reporting more consistent worries at both items.

#### 3.2.8. Economic burden

With regard to the cost of treatments described in the PFQ, a high proportion of caregivers failed to report any expenses over the previous 12 months in their questionnaires. No direct or indirect costs were reported by caregivers living in Southern Italy.

##### 3.2.8.1. Costs for professionals (psychiatrists, neurologists, psychologists, nurses) and other services (except drugs) over the last 12 months with no reimbursement

One hundred and eleven caregivers (81.6%) reported no expenses related to this item. The remaining 25 respondents reported a wide range of costs (from 20 to 13,500 euros) with a mean value of 932 euros (SD 2,629).

##### 3.2.8.2. Costs for alternative medicines, “magicians,” and healers over the last 12 months

Only 3 caregivers (2.2%) reported having incurred expenses in this area, with a limited range of expenditure (200–600 euros) and a mean value of 400 euros (SD 200).

##### 3.2.8.3. Costs for drugs out of reimbursement in the last 12 months

Forty caregivers (29.4%) answered this item, with expenditure ranging from 10 to 1200 euros and a mean value of 585 euros (SD 779).

##### 3.2.8.4. Loss of income over the last 12 months

Almost 90% of caregivers (122) reported no loss of income due to reduced working hours of a family member or having been forced to take a lower paid job. The remaining 14 caregivers (10,3%) indicated a mean yearly loss of 2,285 euros (SD 2528) (range 300–10,000 euros).

#### 3.2.9. Personal growth of caregivers

Item 31 of the FPQ (“*All things considered, I learned something positive from this situation, for example, to understand myself and others better*”) was taken as representing the most important indicator of PG amongst caregivers. Sixty-four caregivers (47.1%) provided a very positive (7.4%) and a positive answer (39.7%), respectively. Sixty-one caregivers were doubtful (44.9%), and only 11 caregivers (8.1%) answered that the illness of their family member had been a highly negative experience from which nothing could be learned.

On assigning a dichotomic value to item scoring (very positive/positive answer vs. doubtful/negative answer), no statistically significant differences were found based on gender and role. A statistically significant distribution was found for geographic location, emphasizing how caregivers living in Southern (53.7%) Italy displayed an increased awareness of the illness of their affected member compared to caregivers living in Central (21.4%) and Northern Italy (21.4%) (chi-square: 9.297; *p* = 0.010).

### 3.3. Correlation between caregiver’s personal growth, family functioning, and burden of care

Table 5 shows the correlations between caregivers’ PG and the three dimensions of the FFQ and burden of care, as measured by FPQ and duration of illness of the affected person.

**TABLE 5:** Correlations between caregivers’ personal growth and the 3 dimensions of FFQ and 6 dimensions of burden of care, as measured by FPQ, and the duration of user illness and relative’s years of education.

		Personal growth	Problem-solving	Communication	Personal goals	Objective burden of care	Subjective burden of care	(Lack of) professional support	(Lack of) family Support	(Absence of) positive comments	Negative comments	User length of illness	Relative’s years of education
Problem-solving	Pearson correlation	−0.383**	–										
	Sign. (two-tailed)	0.000											
Communication	Pearson correlation	−0.270**	0.644**	–									
	Sign. (two-tailed)	0.001	0.000										
Personal goals	Pearson correlation	−0.260**	0.548**	0.459**	–								
	Sign. (two-tailed)	0.002	0.000	0.000									
Objective burden of care	Pearson correlation	0.145	−0.230**	−0.024	−0.390**	–							
	Sign. (two-tailed)	0.092	0.007	0.78	0.000								
Subjective burden of care	Pearson correlation	0.037	−0.192*	0.089	−0.332**	0.648**	–						
	Sign. (two-tailed)	0.67	0.025	0.302	0.000	0.000							
(Lack of) professional support	Pearson correlation	0.179*	−0.399**	−0.344**	−0.196*	0.123	0.148	–					
	Sign. (two-tailed)	0.037	0.000	0.000	0.022	0.155	0.085						
(Lack of) family support	Pearson correlation	0.286**	−0.421**	−0.252**	−0.208*	0.253**	0.262**	0.501**	–				
	Sign. (two-tailed)	0.001	0.000	0.003	0.015	0.003	0.002	0.000					
(Absence of) positive comments	Pearson correlation	0.237**	−0.445**	−0.268**	−0.134	0.282**	0.234**	0.221**	0.337**	–			
	Sign. (two-tailed)	0.006	0.000	0.002	0.121	0.001	0.006	0.01	0.000				
Negative comments	Pearson correlation	−0.022	0.022	0.046	0.024	−0.033	0.116	0.074	0.121	−0.106	—		
	Sign. (two-tailed)	0.8	0.803	0.591	0.779	0.699	0.179	0.391	0.162	0.222			
User length of illness	Pearson correlation	−0.142	0.112	0.159	−0.082	0.047	0.172*	−0.093	−0.026	0.002	−0.046	–	
	Sign. (two-tailed)	0.1	0.194	0.064	0.344	0.591	0.045	0.281	0.76	0.981	0.595		
Relative’s years of education	Pearson correlation	0.11	−0.008	0.119	0.226**	0.011	−0.088	0.018	0.03	−0.038	−0.034	−0.157	–
	Sign. (two-tailed)	0.204	0.928	0.166	0.008	0.901	0.307	0.832	0.733	0.663	0.695	0.068	

Bold values indicate a statistically significant correlation with a *p*-value < 0.05. *p = 0.05; **p = 0.01.

Statistically significant negative correlations were found between caregiver’s PG (in the FPQ instrument, higher scores are associated with a worse evaluation) and the 3 dimensions of problem-solving, communication, and pursuit of personal goals, as measured by FFQ, highlighting how appreciation of the affected person’s illness was associated with better family functioning. Correlation analyses revealed a significant positive correlation between caregiver’s PG and FPQ scores for positive attitudes toward the affected person and support received from professionals, relatives and friends.

Problem-solving skills showed an inverse statistically significant correlation with positive comments relating to the affected person, family, and professional support, as well as to objective and subjective burden of care. Better communication skills were negatively correlated with professional and family support and a positive communication style. The pursuit of reaching individual goals showed an inverse statistically significant correlation with objective and subjective burden of care and professional and family support and a significant positive correlation with caregiver’s years of education.

Length of illness was positively correlated with subjective burden of care of the family member.

### 3.4. Variables impacting on caregiver’s personal growth

Table 6 shows the results of multinominal logistic regression for item 31 of the Questionnaire for Family Problems, Caregiver’s PG, as dependent variable.

**TABLE 6:** Logistic multinominal regression for a = item 31 [“*All things considered, I learned something positive from this situation (for example, to understand myself and others better*,” score 1 D very much)] of the questionnaire for family problems, as dependent variable.

		B	Standard error	Wald	df	Sign.	Exp(B)	95% confidence interval for Exp(B)	
								Lower bound	Upper bound
1. Positive	Intercept	5.304	6.908	0.59	1	0.443			
	Caregiver age	0.037	0.038	0.944	1	0.331	1.038	0.963	1.119
	Caregiver years of education	0.19	0.145	1.731	1	0.188	1.21	0.911	1.606
	User age	0.003	0.088	0.001	1	0.971	1.003	0.844	1.193
	User length of illness	−0.025	0.09	0.077	1	0.781	0.975	0.818	1.163
	Problem-solving	−0.285	0.157	3.285	1	0.07	0.752	0.552	1.023
	Communication	0.098	0.243	0.163	1	0.687	1.103	0.685	1.775
	Personal goal	−0.112	0.171	0.428	1	0.513	0.894	0.639	1.25
	Objective burden of care	−0.067	0.104	0.421	1	0.516	0.935	0.763	1.145
	Subjective burden of care	0.011	0.169	0.004	1	0.948	1.011	0.726	1.409
	Professional support	1.266	1.154	1.205	1	0.272	3.548	0.37	34.037
	Relatives and friends support	−0.335	0.771	0.189	1	0.664	0.715	0.158	3.242
2. Modestly positive	Intercept	3.9	7.016	0.309	1	0.578			
	Caregiver age	0.08	0.041	3.734	1	0.053	1.083	0.999	1.174
	Caregiver years of education	0.214	0.149	2.071	1	0.15	1.239	0.925	1.66
	User age	−0.04	0.092	0.189	1	0.664	0.961	0.803	1.15
	User length of illness	0.018	0.093	0.037	1	0.847	1.018	0.848	1.222
	Problem-solving	−0.347	0.162	4.561	1	**0.033**	**0.707**	0.514	0.972
	Communication	0.138	0.244	0.319	1	0.572	1.148	0.711	1.852
	Personal goal	−0.126	0.177	0.513	1	0.474	0.881	0.624	1.246
	Objective burden of care	−0.031	0.106	0.087	1	0.769	0.969	0.787	1.193
	Subjective burden of care	−0.134	0.178	0.571	1	0.45	0.874	0.617	1.239
	Professional support	1.205	1.191	1.024	1	0.312	3.336	0.323	34.427
	Relatives and friends support	0.071	0.798	0.008	1	0.929	1.074	0.225	5.125
3. Total disagreement	Intercept	−0.734	9.127	0.006	1	0.936			
	Caregiver age	0.053	0.061	0.744	1	0.388	1.054	0.935	1.189
	Caregiver years of education	0.186	0.188	0.977	1	0.323	1.205	0.833	1.742
	User age	0.016	0.123	0.017	1	0.897	1.016	0.799	1.292
	User length of illness	−0.136	0.132	1.057	1	0.304	0.873	0.674	1.131
	Problem-solving	−0.133	0.217	0.373	1	0.542	0.876	0.572	1.341
	Communication	−0.08	0.285	0.079	1	0.779	0.923	0.528	1.614
	Personal goal	−0.19	0.234	0.661	1	0.416	0.827	0.523	1.308
	Objective burden of care	0.137	0.139	0.975	1	0.323	1.147	0.874	1.505
	Subjective burden of care	−0.178	0.251	0.506	1	0.477	0.837	0.512	1.368
	Professional support	−0.543	1.468	0.137	1	0.711	0.581	0.033	10.316
	Relatives and friends support	2.661	1.218	4.773	1	**0.029**	**14.306**	1.315	155.67

^a^The reference category is 1. “All things considered, I learned something positive from this situation, for example, to understand myself and others better”: Higher positive score. Bold values indicate a statistically significant predictor with a *p*-value < 0.05.

The first set of coefficients representing a comparison between caregivers scoring 1 (very positive appreciation of the experience of caregiving) and caregivers scoring 2 (positive milder appreciation) failed to yield any statistically significant predictors.

In the second set of coefficients, representing a comparison between caregivers scoring 1 (very positive appreciation of the experience of caregiving) and caregivers scoring 3 (little appreciation), the only significant negative predictor was total score obtained for “problem-solving,” with caregivers scoring higher on this dimension being less likely to express a highly positive judgment of their experience. Good problem-solving strategies seemed to predict a modest PG of caregivers, whilst not acting as an overt discouragement.

In the third set of coefficients, showing comparison between caregivers scoring 1 (very positive appreciation of the caregiving experience) and caregivers scoring 3 (total disagreement on the opportunity of PG due to the caregiving experience), the only significant positive predictor was “support of relatives and friends.” In this subgroup, caregivers scoring a high level of support from family and friends were 14 times more likely to express a strong appreciation of their caregiving experience.

## 4. Discussion

Caregivers included in our study reported good family functioning, despite a medium-low family socio-economic context and mean length of illness exceeding 20 years in one of their family members. From a psychoeducational perspective, family functioning implies clear and direct communication, efficient problem-solving, and an ability to not become overwhelmed by the illness of a loved one, whilst continuing to work toward one’s own goal to improve the family atmosphere. Less than 15% of caregivers in our sample expressed criticism and hostility toward their affected member, compared to 30% of first-episode psychosis caregivers, who showed depressive symptoms (45). Based on the findings reported by Hamaie et al., the interaction between criticism and caregiver distress may develop during the initial stages of the illness (45). Our study suggests that the longer duration of illness, the extensive caregivers’ experience, and a subsequent better understanding of the illness seem to reduce criticism and hostility. Moreover, more than one-third of our caregivers expressed positive comments and showed warmth and empathetic attitudes, listing the abilities and “talents” of the affected subjects.

Our sample reported a relatively low objective and subjective burden of care, featuring a statistically higher subjective burden for women, adequate support from professionals, and lower level of support from relatives and friends. With regard to sample characteristics, and contrary to previous research (40, 46), no significant relationships were detected in the sample studied between total carer burden and variables such as patient age, patient diagnosis, and patient age at onset of illness (47). Approximately 50% of caregivers from our sample reported PG following the experience of living with a severely mentally ill relative. This result does not seem to find correspondence in the literature, which might display a tendency to overlook similarly positive variables in families with long-term mentally ill members.

Our study confirms the findings of previous research with regard to a direct and close association between good family functioning and low burden of care in caregivers of people affected by schizophrenia. Good problem-solving skills and ability of each family member to pursue their personal goals were both associated with reduced objective and subjective burden, whilst the latter was increased by a lack of support from relatives and friends and scarce positive comments. Although no unambiguous definition for the term’ family functioning’ has been coined to date, several different paradigms coincide. Caregivers of individuals with schizophrenia reporting a low caregiver burden and high levels of family functioning tended to disclose a better quality of life than their counterparts (15). Among other factors, higher family functioning was an important correlate of decreased family burden in high-income countries and low-resource settings (48). High perceived family functioning represented an important buffer and/or protective factor moderating the deleterious effects of psychosis-spectrum symptoms on the role and social functioning of individuals at high clinical risk for psychosis (49). Indeed, the early stages of psychosis are often chaotic and stressful for the affected individuals and their families, resulting in intense family difficulties such as disengagement, rigidity, and chaos compared with normative data from healthy control families (16). Families tend to be more overwhelmed and unsure of how to proceed (40, 50). Moreover, in a clinical sample of youths affected by psychosis-spectrum symptoms, lower family functioning (caregiver-reported) was significantly associated with higher aspects of internalized stigma in these help-seeking subjects (51). Indeed, in our study, the longer caregiving may have contributed to the “adjustment” or “adaptation” of the caregivers to dealing with their affected loved ones and therefore may subjectively (and objectively) experienced lower burden of care.

In our sample, subjects’ length of illness was positively correlated with subjective burden of care perceived by the caregiver. The study confirmed the negative impact of length of illness on family burden. Caregivers of relatives with a longer duration of disease are more likely to experience a subjective burden of care. These findings are consistent with broader empirical evidence reported in recent systematic reviews suggesting the adverse effects of a long duration of psychosis on family members of people with severe mental illness (41, 42). Indeed, the mere perception of a long-term illness seemed to negatively impact caregivers, resulting in more significant distress (43).

The geographical area of residence of our caregivers corresponded to their area of birth and sociocultural background. The distribution of caregivers throughout 3 Italian geographical areas showed modest differences in family functioning: caregivers living in Northern Italy seemed more competent in communication than those living in Central Italy, whilst the latter were apparently less skilled in pursuing their individual goals than caregivers living in Southern Italy. With regard to differences in levels between the three Italian geographical locations, in agreement with the findings of Magliano et al., our study confirmed a homogeneous burden of care, without distinction, in the three Italian geographical areas considered (10). Indeed, Magliano et al. identified a lower burden in Northern Italy, but following a cross-check for psychosocial interventions received, differences in family burden between the three geographical areas were no longer evident (10). Samples recruited in Central and Southern Italy reported greater satisfaction in the support provided by relatives and friends than caregivers living in Northern Italy. The results of this study partially confirmed the hypothesis of an increasingly supportive social network amongst the general population in the South (10) compared to a sample living in Northern Italy. Indeed, Northern Italian families may resemble more Western European families compared to the more traditional families in the South. In fact, over the last 50 years, the regions of Southern Italy have been characterized by the highest percentage of large families, while Northern Italy accounts for the highest rate of single-member households (52).

Our findings align with those of a series of previous publications investigating the relevance of social support in reducing family burden in schizophrenia (10, 15, 44, 53–55) and impacting on caregivers wellbeing (5). Likewise, social support was reported as the second-highest predictor of quality of life in a Spanish sample studied by Ribè et al. (15). In our sample, support provided by relatives and friends appeared to represent the strongest variable influencing full appreciation of the caregiving experience and PG, thus increasing by more than 14-fold the likelihood of positive appreciation of the caregiving experience.

Personal growth in caregivers was associated with good family functioning and adequate support from professionals, relatives and friends. The aspect defined in the present study as “Personal Growth” of the caregiver was similar to the content of the Caregiving Rewarding Feelings (CRF) scale applied in a Chinese sample to evaluate positive feelings amongst primary caregivers, i.e., “*become more loving and patient*,” “*feels more worthy*,” “*be more active and optimistic*,” “*have a stronger sense of responsibility*” (56). Zhou et al. found that rewarding feelings amongst caregivers were positively associated with understanding schizophrenia and mastering caregiving skills (56). Jansen et al. reported how caregivers describing more positive caregiving experiences possessed greater levels of metacognition (57), i.e., a greater capacity to form complex ideas about oneself and others and a more balanced perspective of caregiving, in which there is room for both positive and negative experiences. Gupta and Bowie (16) found that positive caregiver appraisals emerged as the only significant predictor of family functioning. However, this only accounted for 9% of variance, suggesting that other variables not measured in their study accounted for a substantial portion of family functioning in their population (16). A very recent qualitative study explored the long-term experiences of family caregivers who had been looking after their affected family members for more than 20 years. The authors reported how the majority of caregivers expressed positive thoughts and, influenced by the cultural values of rural Chinese familism, claimed to be satisfied with their lives, arguing that family members are expected to care for each other (58). In the present study, we likewise estimated the presence of an extended Italian familism involving relatives and friends, particularly in Southern Italy, where families are frequently larger. The findings obtained in our study highlighted how caregivers living in Southern Italy were able to better appreciate the experience of illness of their affected family member than caregivers residing in Central and Northern Italy.

Accordingly, our initial hypothesis relating to variables capable of promoting caregiver’s PG, intended as a positive experience in assisting a person affected by severe mental illness, was only partially confirmed by our estimated comprehensive psychosocial model. When good family functioning was confirmed as being negatively correlated with burden of care which, in turn was linked to a longer duration of illness, variables identified as contributing toward the PG of caregivers included the voicing of positive comments referred to the affected relative together with the support of relatives and friends, but not young age of the caregiver. However, the only variable apparently capable of strongly influencing caregivers’ PG was represented by the support of relatives and friends, thus likely helping caregivers to overcome the sense of isolation created by assuming sole responsibility in care of the ill relative.

### 4.1. Strengths and limitations

To the best of our knowledge, this is the first multicentric Italian study to evaluate positive aspects of the caregiving experience of living with people affected by schizophrenia.

Four main limitations of the present study should, however, be acknowledged. Firstly, this add-on protocol study recruited a limited number of the caregivers taking part in the main protocol study involving 921 mentally ill subjects and 342 unaffected relatives. The Italian NIRP Multicenter Study was a highly articulated study conducted to investigate the effect of illness-related variables, personal resources, and context-related factors on real-life functioning of people with schizophrenia (35). Despite their interest, not all centers were able to participate in this add-on protocol due to professional resource issues.

Secondly, length of illness was the sole illness-related variable included in both our study and the main study, with the latter focusing more on the impacts of chronic care than on variables of psychopathological and social functioning.

Third, the study did not take into consideration concurrent positive and protective factors for caregivers beyond family functioning and burden of care (e.g., cultural and socio-economic factors).

Fourth, the level of burden of care may also have been attributable to factors related to the delivery of health services (41). In our study, we can reasonably assume that the nine University centers involved in this research delivered homogenous standards and levels of care.

## 5. Conclusion

The caregiving experience is capable of influencing both the course of illness of the affected person and wellbeing of their caregivers. In assisting a loved one on their long journey through mental illness, caregivers are faced with unique challenges, often aware that any expectations and aspirations for their ill relative are unlikely to be realized whilst, at the same time, trying to remain hopeful and optimistic. Beyond the objective commitment and emotional suffering produced by having a family member affected by severe mental illness, effective family skills may contribute to offsetting the burden of care. The numerous challenges and positive aspects associated with caregiving should be duly acknowledged by mental health services and integrated into routine clinical assessment and intervention framework.

## Data availability statement

The raw data supporting the conclusions of this article will be made available by the authors, without undue reservation.

## Ethics statement

The studies involving human participants were reviewed and approved by the Ethical Committee University of L’Aquila (2014). The patients/participants provided their written informed consent to participate in this study.

## Author contributions

RR and SG had full access to all the data in the study and they took responsibility for the data’s integrity and the data analysis’s accuracy and contributed to the study supervision. RR, SG, and MC contributed to the study concept and design. LG, VB, BC, EA, MA, SB, AB, PB, GC, CC, AF, CM, AM, FP, and AS contributed to the collection, analysis, and interpretation of data. All authors contributed to the critical revision of the manuscript and agreed to be responsible for all aspects of the work in ensuring that questions relating to the accuracy or integrity of any part of the work were adequately reviewed and resolved, read, and manuscript approved.
